# A lost opportunity for science: journals promote data sharing in metabolomics but do not enforce it

**DOI:** 10.1007/s11306-017-1309-5

**Published:** 2017-12-26

**Authors:** Rachel A. Spicer, Christoph Steinbeck

**Affiliations:** 1 European Molecular Biology Laboratory, European Bioinformatics Institute (EMBL-EBI), Wellcome Trust Genome Campus, Hinxton, Cambridge CB10 1SD UK; 2 Institute for Inorganic and Analytical Chemistry, Friedrich-Schiller-University, Jena, Germany

**Keywords:** Data sharing, Open data, Metabolomics, Journal

## Abstract

**Introduction:**

Data sharing is being increasingly required by journals and has been heralded as a solution to the ‘replication crisis’.

**Objectives:**

(i) Review data sharing policies of journals publishing the most metabolomics papers associated with open data and (ii) compare these journals’ policies to those that publish the most metabolomics papers.

**Methods:**

A PubMed search was used to identify metabolomics papers. Metabolomics data repositories were manually searched for linked publications.

**Results:**

Journals that support data sharing are not necessarily those with the most papers associated to open metabolomics data.

**Conclusion:**

Further efforts are required to improve data sharing in metabolomics.

## Introduction

The concepts of data sharing and open data are becoming increasingly important in science. Areas as diverse as psychology (Open Science Collaboration 2015), medicine (Begley and Ellis 2012) and computer science (Collberg and Proebsting 2016) have been affected by the ‘replication crisis’ and two-thirds of scientists report being concerned about reproducibility (Reality check on reproducibility 2016). Sharing data publicly is an important way of improving reproducibility and showing that researchers are confident in their work (McKiernan et al. 2016). Studies with data shared in a repository also receive more citations than those without publicly available data (Piwowar and Vision 2013).

As more funding bodies, journals and societies are now encouraging or mandating data sharing, it is important to look at the effectiveness of these policies. In this study we review the data sharing policies of the journals with publications associated with the most publicly available metabolomics data.

## Data sharing in metabolomics

**Fig. 1:**
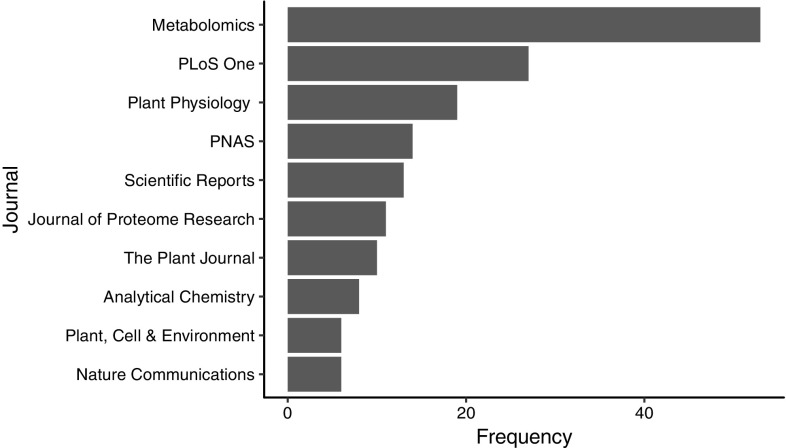
The ten journals with the highest frequency of publications directly linked from a publicly available metabolomics study, in a dedicated repository (MetaboLights, Metabolomics Workbench, MetaPhen, MeRy-B and GNPS)

There are now > 750 metabolomics studies indexed on MetabolomeXchange (http://www.metabolomexchange.org/) and > 1300 on OmicsDI (Perez-Riverol et al. 2017). These studies with data publicly available in dedicated repositories [MetaboLights (Haug et al. 2013), Metabolomics Workbench (Sud et al. 2016), MetaPhen (Carroll et al. 2015), MeRy-B (Ferry-Dumazet et al. 2011) and GNPS (Wang et al. 2016)], directly link to 368 unique journal articles (as of 15th September 2017). 45.4% of these 368 journal articles are published in ten journals (Fig. 1), with 58 (14.4%) being published in the Metabolomics journal.

Of these 10 journals, PLoS One, PNAS, Scientific Reports and Nature Communications require data availability statements to be included within submitted manuscripts. PLoS One specifically requires data be submitted to an appropriate public repository, whereas Nature Journals (Nature Communications and Scientific Reports) and PNAS only encourage it. Metabolomics and Plant Physiology require that authors make materials available to investigators for non-commercial research purposes, with Metabolomics specifying raw data must be shared and suggesting users deposit their data in a repository.

**Fig. 2:**
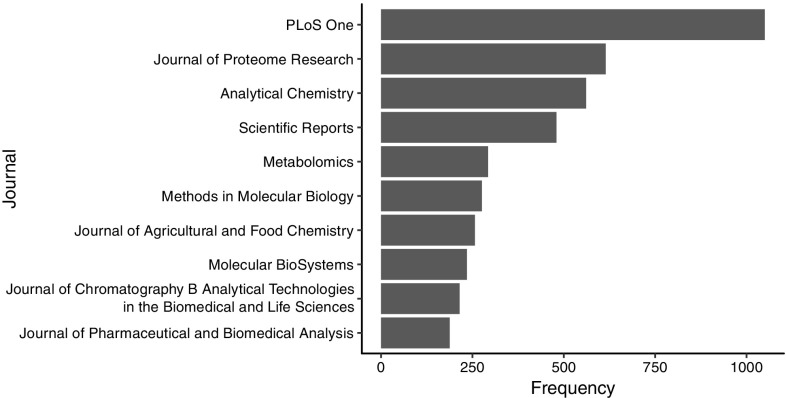
The ten journals with the highest frequency of publications when searching PubMed for “metabolome” OR “metabolomics”

There are more than 17,000 journal articles on metabolomics indexed in PubMed (when searching for “metabolome” OR “metabolomics”). The ten journals that have published the highest number of papers returned when searching PubMed using this criteria are shown in Fig. 2. Whilst PLoS One appears to have the highest number of metabolomics papers, it is worth noting that only ~ 25% (304/1178) of articles published in the journal Metabolomics are indexed in PubMed.

Searching PubMed like this provides only a very rough estimate of the total number of metabolomics journal articles, as metabolomics papers may not contain the words “metabolome” or “metabolomics” in their title or abstract, may not be indexed by PubMed, or non-metabolomics papers may be returned. In fact, 44% of publications directly associated with metabolomics studies are not returned when searching for “metabolome” OR “metabolomics” in PubMed. Of these, 12.6% were not indexed on PubMed and 31.8% were indexed, but not returned. Despite this, it can be assumed that the majority of articles returned when searching for “metabolome” OR “metabolomics” are of metabolomics research.

Two of the journals with highest number of metabolomics papers on PubMed have no data sharing policies (Analytical Chemistry and Methods in Molecular Biology). The Journal of Proteome Research encourages users to deposit proteomics data in ProteomeXchange (Vizcaíno et al. 2014), however, unsurprisingly, has no policy on metabolomics data. Molecular BioSystems, the Journal of Chromatography B and the Journal of Pharmaceutical and Biomedical Analysis all encourage data sharing.

Given the number of journal articles published in the field of metabolomics, it would be expected that far more studies make their data open than actually do. The current data sharing policy of PLoS One has been in place since March 2014 (Bloom et al. 2014) and Springer Nature have had their policy since September 2016 (Announcement: Where are the data? 2016). Since 2015 PLoS One has published ~ 400 metabolomics papers and since 2017 Scientific Reports has published > 140. Despite not requiring data sharing via a dedicated repository, articles published by the metabolomics journal share data in dedicated repositories at a higher rate than those in PLoS One.

Although MetaboLights, is one of PLoS One’s recommended repositories for omics data, users may remain unaware of dedicated metabolomics repositories and instead publish their data in general repositories such as Dryad (https://datadryad.org/), figshare (https://figshare.com/) or Zenodo (https://zenodo.org/). As, PLoS One’s data sharing policy specifically states “authors do not need to submit the raw data collected during an investigation if the standard in the field is to share data that have been processed”, authors may feel that metabolomics is one such field where sharing only preprocessed data or an annotated list of identified metabolites is sufficient, rather than raw spectral data.

Another possibility is that journals such as PLoS One and Scientific Reports publish a higher percentage of clinical research or other studies with human participants. Due to concerns of patient privacy and consent, both journals have different data sharing requirements for clinical studies compared to those for studies including non-human subjects. Only summary, rather than raw data, must be reported for clinical studies.

The Ethical, Legal and Social Implications (ELSI) of sharing data from research involving human participants should always be considered, and protecting patient privacy must be a priority. However, except potentially in the case of rare diseases, there is currently no known means of identifying a patient from their metabolic profile. This is especially true for large cohort studies that include many patients with the same disease. There is a far greater risk of patient identification from genetic data than metabolomic. Despite this, a repository for sensitive genomics data has been developed, the European Genome-phenome Archive (EGA) (Lappalainen et al. 2015), which allows controlled access to datasets that cannot be made publically available. A similar repository could be established for clinical metabolomics data, allowing researchers to identify studies containing data relevant to their research by searching for diseases, metabolites or pathways of interest (metadata). Users could then apply to the studies’ data access committee for access to the dataset of interest. As recommended by the H2020 PhenoMenAl guidelines (http://phenomenal-h2020.eu/home/) for encoding data terms of use in the ISA format (and adopted by EGA), metadata describing terms of use, consent availability and additional ancillary information in the repository should be encoded using the Data Use Ontology (https://www.ebi.ac.uk/ols/ontologies/duo).

An additional concern is the number of publicly available metabolomics studies with raw data that have no associated publication: > 800. Associated journal articles probably exist for much of this open data, however there is no direct link between the data and the literature. This hinders the re-use of data, as papers likely contain more detailed experimental design descriptions and additional metadata, and data alone are insufficient for reanalysis (Kind and Fiehn 2009).

The connection between the publication review process at journals and the deposition of data to public repositories (such as MetaboLights or Metabolomics Workshop) must be improved. Potential methods to enhance this connection include Research Resource Identifiers (RRID) and project preregistration. RRID are unique, persistent identifiers that can be used for referencing a research resource, such as software, organisms or cell lines. Publishers could use RRID to link publications to data. Following the generation of the experimental design, projects can be preregistered—outlining what data and analysis will be performed prior to observing the research outcomes. Examples of repositories that allow project preregistration include the European Bioinformatics Institute’s BioSamples (Faulconbridge et al. 2014) and the National Center for Biotechnology Information’s BioProject (Barrett et al. 2012).

For metabolomics to become an established clinical tool, meta-analyses must be performed. This is necessary in order to demonstrate that quantitative metabolite measurements are reliable and accurate across studies. Meta-analysis cannot be performed without available data. It is also worth noting that data are valuable research outputs in their own right. However, a cultural shift is required to citing data themselves, rather than citing journal articles, in order to give data their full credit. The metabolomics community must move away from accepting non-interoperable summary tables as an acceptable way of disseminating data and towards requiring data sharing.

## Conclusion

Metabolomics is still lagging behind other omics in regards to data sharing. The value of open data has been demonstrated in transcriptomics and proteomics where there are many examples of data reuse (Rung and Brazma 2013; Vaudel et al. 2016). In metabolomics, journals that most support data sharing are not necessarily those with the highest number of papers associated to open metabolomics data. In more mature communities such as genomics, it has now become the absolute default that data must be shared. There is hope that time will lead to a similar situation in metabolomics. One positive next step would be to move from encouraging to requiring data sharing and to demand deposition in community-accepted repositories rather than providing potentially vague and open-to-interpretation data sharing guidelines. A metabolomics equivalent repository to EGA could be established to allow for controlled sharing of clinical data, addressing ethics and privacy concerns. There must also be greater effort to improve the linking of data to publications and vice versa.

## Data availability statement

The datasets generated during and/or analysed during the current study, along with the analysis code are available on the GitHub https://github.com/RASpicer/Metabolomics_Data_Sharing. All of the analysis was performed using R version 3.3.2.
